# Impaired Spontaneous Baroreceptor Reflex Sensitivity in Patients With COPD Compared to Healthy Controls: The Role of Lung Hyperinflation

**DOI:** 10.3389/fmed.2021.791410

**Published:** 2022-01-03

**Authors:** Anna Katharina Mayr, Victoria Wieser, Georg-Christian Funk, Sherwin Asadi, Irene Sperk, Matthias Helmut Urban, Arschang Valipour

**Affiliations:** ^1^Karl Landsteiner Institute for Lung Research and Pulmonary Oncology, Vienna, Austria; ^2^Department of Internal and Respiratory Medicine, Klinik Floridsdorf, Vienna, Austria; ^3^Department of Emergency Medicine, Klinik Hietzing, Vienna, Austria; ^4^Department of Internal and Respiratory Medicine, Klinik Ottakring, Vienna, Austria; ^5^Department of Pediatrics, Klinik Donaustadt, Vienna, Austria

**Keywords:** COPD, chronic obstructive pulmonary disease, lung hyperinflation, baroreceptor reflex sensitivity, BRS, autonomic nervous system, cardiovascular disease, cardiopulmonary interaction

## Abstract

**Background and Objectives:** Patients with chronic obstructive pulmonary disease (COPD) are at increased risk for cardiovascular disease. This study aimed to investigate the relationship between pulmonary hyperinflation and baroreceptor reflex sensitivity (BRS), a surrogate for cardiovascular risk.

**Methods:** 33 patients with COPD, free from clinical cardiovascular disease, and 12 healthy controls were studied. Participants underwent pulmonary function and non-invasive hemodynamic measurements. BRS was evaluated using the sequence method during resting conditions and mental arithmetic stress testing.

**Results:** Patients with COPD had evidence of airflow obstruction [forced expiratory volume in 1 s predicted (FEV_1_%) 26.5 (23.3–29.1) vs. 91.5 (82.8–100.8); *P* < 0.001; geometric means (GM) with 95% confidence interval (CI)] and lung hyperinflation [residual volume/total lung capacity (RV/TLC) 67.7 (64.3–71.3) vs. 41.0 (38.8–44.3); *P* < 0.001; GM with 95% CI] compared to controls. Spontaneous mean BRS (BRSmean) was significantly lower in COPD, both during rest [5.6 (4.2–6.9) vs. 12.0 (9.1–17.6); *P* = 0.003; GM with 95% CI] and stress testing [4.4 (3.7–5.3) vs. 9.6 (7.7–12.2); *P* < 0.001; GM with 95% CI]. Stroke volume (SV) was significantly lower in the patient group [−21.0 ml (−29.4 to −12.6); *P* < 0.001; difference of the means with 95% CI]. RV/TLC was found to be a predictor of BRS and SV (*P* < 0.05 for both), independent of resting heart rate.

**Conclusion:** We herewith provide evidence of impaired BRS in patients with COPD. Hyperinflation may influence BRS through alteration of mechanosensitive vagal nerve activity.

## Introduction

Chronic obstructive pulmonary disease (COPD) is characterized as a common, preventable disease of the respiratory system, often associated with various extrapulmonary comorbidities like cardiovascular diseases (1, 2). Previous studies reported evidence of a direct interaction between the hyperinflated lungs and the heart, with elevated lung volumes leading to consecutive impairment in cardiac filling (3–5).

Moreover, there is evidence of impaired autonomic function and baroreceptor reflex sensitivity (BRS) in patients with COPD (6–9). Impaired BRS in turn is considered as an independent prognostic factor in patients with coronary heart disease (10).

Thus, the aim of the current study was to evaluate the relationship between hyperinflation and BRS in patients with stable COPD. An impairment in BRS compared to healthy controls with further deterioration concomitant to increases in airflow obstruction and hyperinflation was hypothesized.

## Methods

### Participants

For this observational case-control study, we examined patients between 40 and 75 years with elevated lung volumes and signs of hyperinflation due to emphysematous type of severe or very severe COPD, defined as a forced expiratory volume in one second predicted (FEV_1_%) of <50% according to GOLD criteria and a residual volume/total lung capacity (RV/TLC) of at least 50%, compared to healthy individuals (1).

Patients were recruited from our outpatient clinic, undergoing evaluation for bronchoscopic lung volume reduction. They had to provide high-resolution computed tomography scan as well as echocardiography not older than 6 months and report a history of stable COPD, defined as a disease without exacerbations and changes in respiratory medication during the previous 4 months. Exclusion criteria furthermore were a history of and medication for any cardiovascular comorbidities, severe pulmonary hypertension or diabetes mellitus, a diffusion capacity of the lung for carbon monoxide (DLCO) <20%, giant bullae in the provided computer tomography, alpha-1-antitrypsin deficiency, prior thoracotomy, excessive sputum production, hypercapnia with an arterial partial pressure for carbon dioxide (paCO_2_) >55 mmHg and a body-mass-index (BMI) >35. All patients had to be ex-smokers with abstinence from smoking for at least 4 months.

Recruitment of healthy, never-smoking individuals of similar age, gender and BMI was achieved during a lung health awareness campaign day. These subjects were included only in the absence of any history or symptoms of or medication for pulmonary or cardiac diseases.

All participants underwent detailed medical and medication history, physical examination, arterial blood gas analysis, post-bronchodilator spirometry and bodyplethysmography according to ATS/ERS standards (11). Normal values were estimated using the reference equations of the European Respiratory Society (12). Lung function measurements were performed with the MasterScreen Body™, Jaeger, Germany. Measurement of DLCO was only performed in patients with COPD, not in the healthy population.

According to regulations of the Ethics Committee of the city of Vienna, the Institutional Review Board approved the study and all participants gave informed consent. The study was conducted in accordance with the Declaration of Helsinki.

### Setting

All cardiovascular studies were performed during the same time of the day to avoid potential circadian variations in measurements of parameters. Alcohol, coffee and tea were prohibited for at least 12 h and heavy exercise for at least 24 h prior to the testing sessions. On the study day, participants were taken to a quiet, dimly-lit room. They rested in supine position on a comfortable bed for at least 20 mins to stabilize cardiovascular parameters before starting the measurements. Thereafter, a 15 min recording of baseline parameters was obtained during quiet normal breathing, which was followed by mental arithmetic stress testing (13). In the patient group, assessment of hemodynamic measurements and BRS was performed after regular inhaler use to align with post-bronchodilator spirometry results.

### Measurements

Continuous measurements of systolic (BPsys) and diastolic (BPdia) beat-to-beat arterial blood pressure were obtained by using the vascular unloading technique of the finger (Task Force® Monitor, CNSystems, Graz, Austria) (14). Mean arterial blood pressure (BPmean) was obtained by integration of the digital pressure waveform. Beat-to-beat blood pressure values were automatically corrected to an offset obtained from oscillometric blood pressure measurements at the brachial artery of the contralateral arm. Real-time beat-to-beat stroke volume (SV) was derived using an improved method of transthoracic impedance cardiography (Task Force® Monitor, CNSystems, Graz, Austria) (15).

BRS was evaluated by the Task Force® Monitor using the sequence method (16–18). This method is based on computer identification of spontaneously occurring sequences in which progressive increases in BPsys of at least 1 mmHg/beat for at least three consecutive heart beats are followed with an one-beat delay by a progressive lengthening in pulse interval (PI) of at least 4 ms/beat (up-sequences) or, *vice versa*, progressive decreases in BPsys followed by a progressive shortening in PI (down-sequences). The slope of each regression line between BPsys and PI changes was taken as an index for the sensitivity of arterial baroreflex modulation of heart rate. Since PI, and not heart rate, is used for calculation, this ensures a positive direction of the slopes, regardless of up- or down-sequences. Only episodes with correlation coefficients greater than 0.95 were selected and from all regression lines a mean slope was calculated to account for BRS, both for up- and down-sequences (BRSup or BRSdown) and for each steady state period (resting condition or mental arithmetic stress testing). The mean of BRSup and BRSdown was taken to indicate overall BRS (BRSmean), again both for resting condition and arithmetic stress testing. A patient example of BRS measurements was provided in the Supplementary Figure 1.

Since an inverse relationship between heart rate and BRS had been described (13), we used prevailing heart rate as an independent variable in multivariable regression analysis to adjust for it.

### Statistics

Statistical analyses were calculated using SPSS® (version 23, SPSS, IBM Corporation, Armonk, USA). Figures were made using GraphPad Prism® (version 5; GraphPad, Software Inc., La Jolla, USA). There was no formal sample size calculation. The number of patients and controls was chosen pragmatically.

For descriptive statistics, variables were assessed for normal distribution using normal plots, whereby logarithmic normally distributed variables (FEV_1_/FVC, FEV_1_%, RV/TLC, FRC, BRSup, BRSdown, BRSmean) were logarithm-transformed. Normally distributed variables were indicated as arithmetic means, logarithmic normally distributed variables as geometric means after back transformation, both with their 95% confidence interval (CI).

Comparative statistics between the groups were made using paired or unpaired t-tests. The mean differences with 95% CI were calculated for normally distributed variables. Comparison of logarithmic normally distributed variables was achieved by using the t-test after logarithmic transformation. After back transformation of the logarithmic arithmetic means as well as the mean differences with their 95% CI, values were indicated as geometric means and ratios of geometric means, each with their 95% CI, as recommended by Bland and Altman for better interpretability of logarithmic normally distributed variables (19, 20). In case of heteroscedasticity, Kruskal-Wallis ANOVA was used. Comparison of categorical variables between the groups was achieved using Fisher's exact test. Bivariable correlations were calculated using Pearson's correlation test. We used multivariable regression analysis to account for heart rate correction of the relationship between lnRV/TLC and SV as well as lnRV/TLC and lnBRSmean and to observe the impact of lnBRSmean and lnRV/TLC on SV. RV/TLC has been selected as the independent variable for multivariable regression analysis in this report, given its previous reported prognostic value for all-cause mortality in COPD (21). A two-sided *P* value of 0.05 was used for all analysis.

## Results

### Baseline Characteristics

104 patients were examined for eligibility. 33 patients had a history of cardiovascular comorbidities, 8 reported deterioration of symptoms with need for oral steroids or antibiotics in the previous 4 months and computed tomography of another 15 patients showed multiple giant bullae, defining them as non-eligible. After examination, another 15 patients were ruled out, 8 because of a DLCO <20% and 7 because of a paCO_2_ >55mmHg. Therefore, 33 patients were included, whereby all completed study examinations.

62 individuals of similar age and BMI were examined for eligibility as controls. 22 had a history of smoking, 24 were on medication for cardiovascular diseases or diabetes, thus defining them as non-eligible. Another 4 individuals were ruled out after examination because of newly-diagnosed airflow obstruction in spirometry. All remaining 12 individuals completed study examinations.

The baseline characteristics of the participants are summarized in Table 1. The study population included more women than men (30 vs. 15), whereby gender proportion was similar between the groups. Patients with COPD had, by definition, significantly more severe airflow obstruction and higher lung volumes compared to controls. Arterial partial pressure of oxygen (paO_2_) was lower in the patient group but paCO_2_ did not differ. All patients were at least on dual long-acting bronchodilator therapy, 31 used triple therapy with inhaled corticosteroids.

**Table 1:** Baseline characteristics are indicated as arithmetic means with 95% confidence intervals (CI), except * as geometric means with 95% CI.

	**COPD (*n* = 33)**	**Healthy subjects (*n* = 12)**	**Difference and 95% CI**	***P value***
Female/male, %	70/30	58/42		0.496
Age, years	60 (45–75)	62 (55–69)	−2 (−6 to 3)	0.479
BMI^§^, kg/m^2^	23 (15–32)	26 (18–33)		0.102
LAMA/LABA	31	0		
LAMA/LABA/ICS	2	0		
FEV_1_/FVC	33.2 (30.4–36.8)*	84.9 (81.0–89.2)*	0.4 (0.3–0.5)^†^	<0.001
FEV_1_, %	26.5 (23.3–29.1)*	91.5 (82.8–100.8)*	0.3 (0.2–0.3)^†^	<0.001
FVC, L	2.1 (1.9–2.3)	3.6 (3.1–4.2)	−1.5 (−2.0 to −1.1)	<0.001
VC, L	2.3 (2.1–2.6)	3.6 (3.1–4.2)	−1.3 (−1.8 to −0.8)	<0.001
VC, %	66.8 (61.7–71.8)	98.0 (90.6–105.4)	−31.2 (−39.9 to −22.5)	<0.001
FRC, L	5.9 (5.2–7.0)*	3.4 (2.7–3.9)*	1.8 (1.3–2.4)^†^	0.001
TLC, L	7.1 (4.5–9.6)	6.2 (3.6–8.8)	0.9 (0–1.8)	0.051
TLC, %	140.3 (102.9–177.8)	103.8 (82.5–125.2)	36.5 (24.6–48.3)	<0.001
RV, L	4.8 (2.8–6.8)	2.6 (1.3–3.8)	2.3 (1.6–2.9)	<0.001
RV/TLC	67.7 (64.3–71.3)*	41.0 (38.8–44.3)*	1.7 (1.5–1.8)^†^	<0.001
DLCO, mmol/min/kPa	4.2 (3.0–5.5)			
DLCO, %	33.8 (18.3–49.2)			
paO_2_, mmHg	68.3 (49.5–87.1)	83.6 (70.1–97.0)	−15.3 (−21.5 to −9.1)	<0.001
paCO_2_, mmHg	41.2 (29.5–52.8)	41.2 (36.4–46.0)	0 (−3.6 to 3.6)	0.999

*Differences are indicated as mean differences between arithmetic means with 95% CI, except*

† *as ratio of the geometric means with 95% CI. Comparison was performed using the unpaired t-test, except*

§ *the Kruskal-Wallis-test. Comparison of gender proportion was made with the Fisher's exact test*.

*COPD, chronic obstructive pulmonary disease; BMI, body mass index; LAMA, long-acting muscarinic antagonists; LABA, long-acting beta agonists; ICS, Inhaled corticosteroids; FEV_1_, forced expiratory volume in one second; FVC, forced vital capacity; VC, vital capacity; FRC, functional residual capacity; TLC, total lung capacity; RV, residual volume; DLCO, diffusion capacity of the lung for carbon monoxide; paO_2_, arterial partial pressure for oxygen; paCO_2_, arterial partial pressure for carbon dioxide; CI, confidence interval*.

### Hemodynamic Measurements

**Figure 1:**
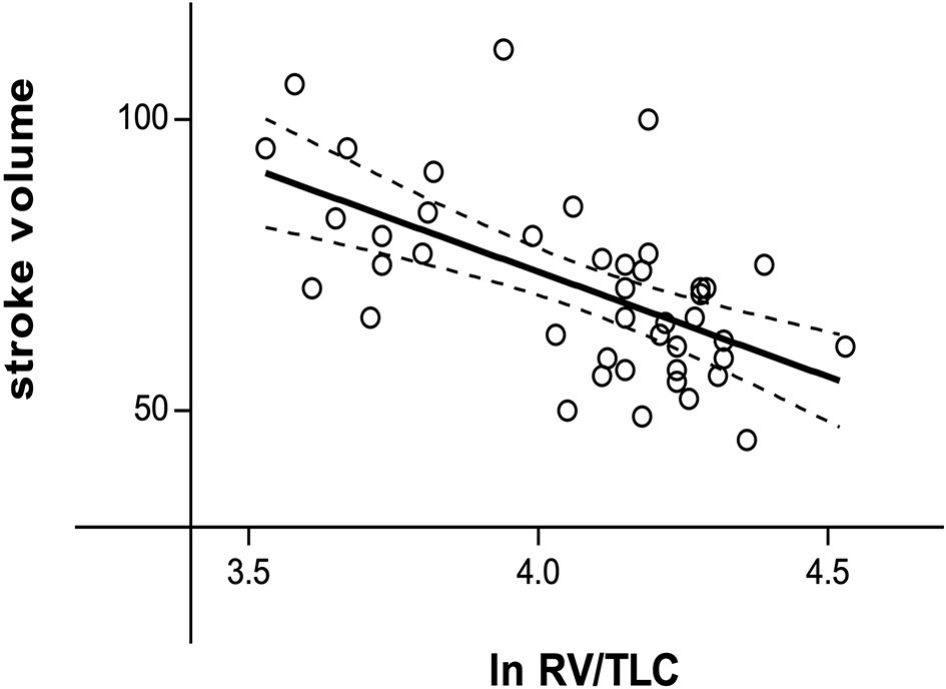
Relationship between the natural logarithm of RV/TLC and stroke volume during resting conditions (*r* = −0.585, *P* < 0.001). ln RV/TLC, natural logarithm of residual volume/total lung capacity.

Patients with COPD had significantly higher heart rate, both during rest and stress, higher blood pressure, lower SV and stroke index (SI) during rest compared to healthy subjects (Table 2). SV and SI during rest correlated significantly with the severity of hyperinflation (lnRV/TLC) (SV: *r* = −0.585, *P* < 0.001, Figure 1; SI: *r* = −0.533, *P* < 0.001). Multivariable regression analysis showed lnRV/TLC to be a predictor of SV, independent of heart rate (β = −0.314, *P* = 0.020).

**Table 2:** Hemodynamic characteristics are indicated as arithmetic means with 95% CI.

	**COPD**	**Healthy subjects**	**Difference and 95% CI**	***P value***
HR rest, bpm	83 (60–107)	67 (49–84)	17 (9–25)	<0.001
HR mental stress, bpm	89 (63–116)	75 (52–97)	15 (6–24)	0.002
BPsys^*^, mmHg	132 (93–171)	118 (101–134)		0.009
BPdia^*^, mmHg	87 (59–114)	77 (64–90)		0.008
SV, ml	65.2 (42.5–88.0)	86.2 (58.6–113.8)	−21.0 (−29.4 to −12.6)	<0.001
SI, ml/m_2_	38.0 (28.5–47.6)	46.4 (34.7–58.0)	−8.3 (−11.9 to −4.8)	<0.001

*Differences are indicated as mean differences between arithmetic means with 95% CI. Comparison was performed using the unpaired t-test, except*

* *the Kruskal-Wallis-test*.

*COPD, chronic obstructive pulmonary disease; HR, heart rate; BPsys, systolic blood pressure; BPdia, diastolic blood pressure; SV, stroke volume during rest; SI, stroke index during rest; CI, confidence interval*.

### Baroreceptor Reflex Sensitivity

**Figure 2:**
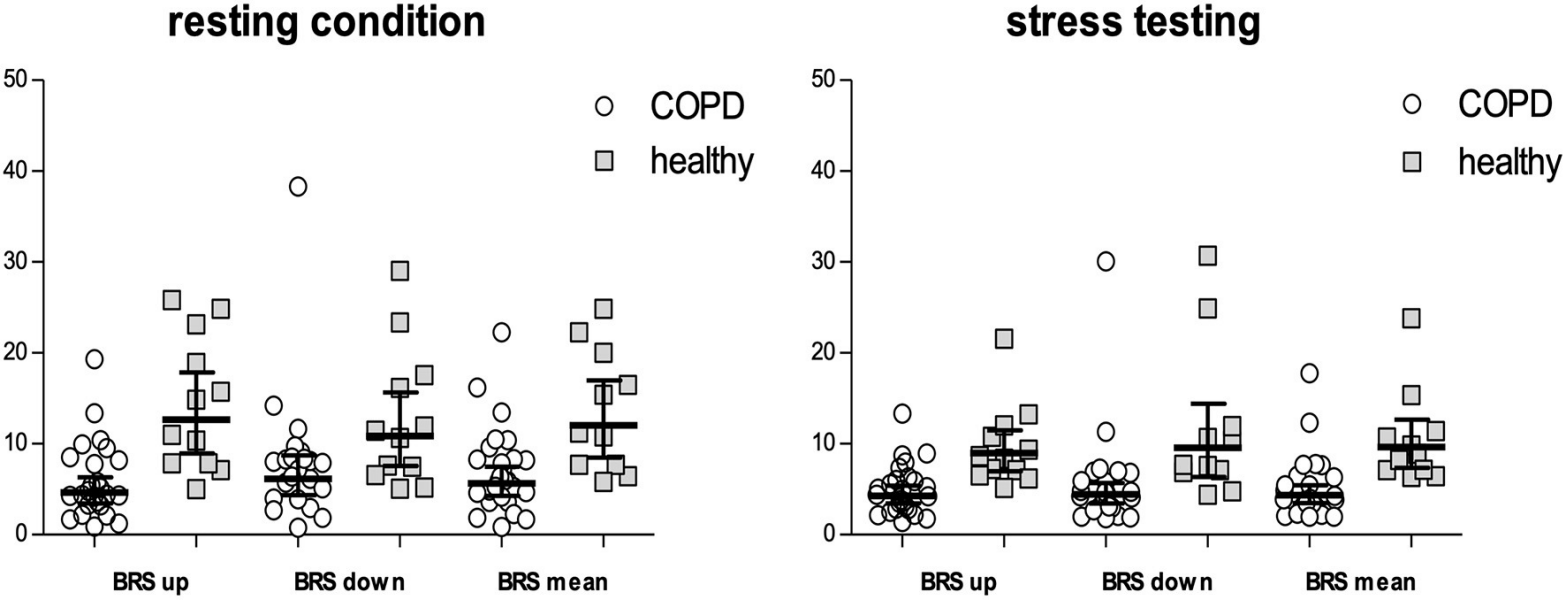
BRSup, BRSdown and BRSmean either during resting conditions as well as mental arithmetic stress testing. Values are indicated as geometric means with 95% CI. Values of COPD patients are shown by circles, values of the healthy population by squares. BRS, baroreceptor reflex sensitivity; BRSup, mean slope of sequences characterized by progressive increase in pulse interval and systolic blood pressure; BRSdown, mean slope of sequences characterized by progressive decrease in pulse interval and systolic blood pressure; BRSmean, mean of BRSup and BRSdown.

**Figure 3:**
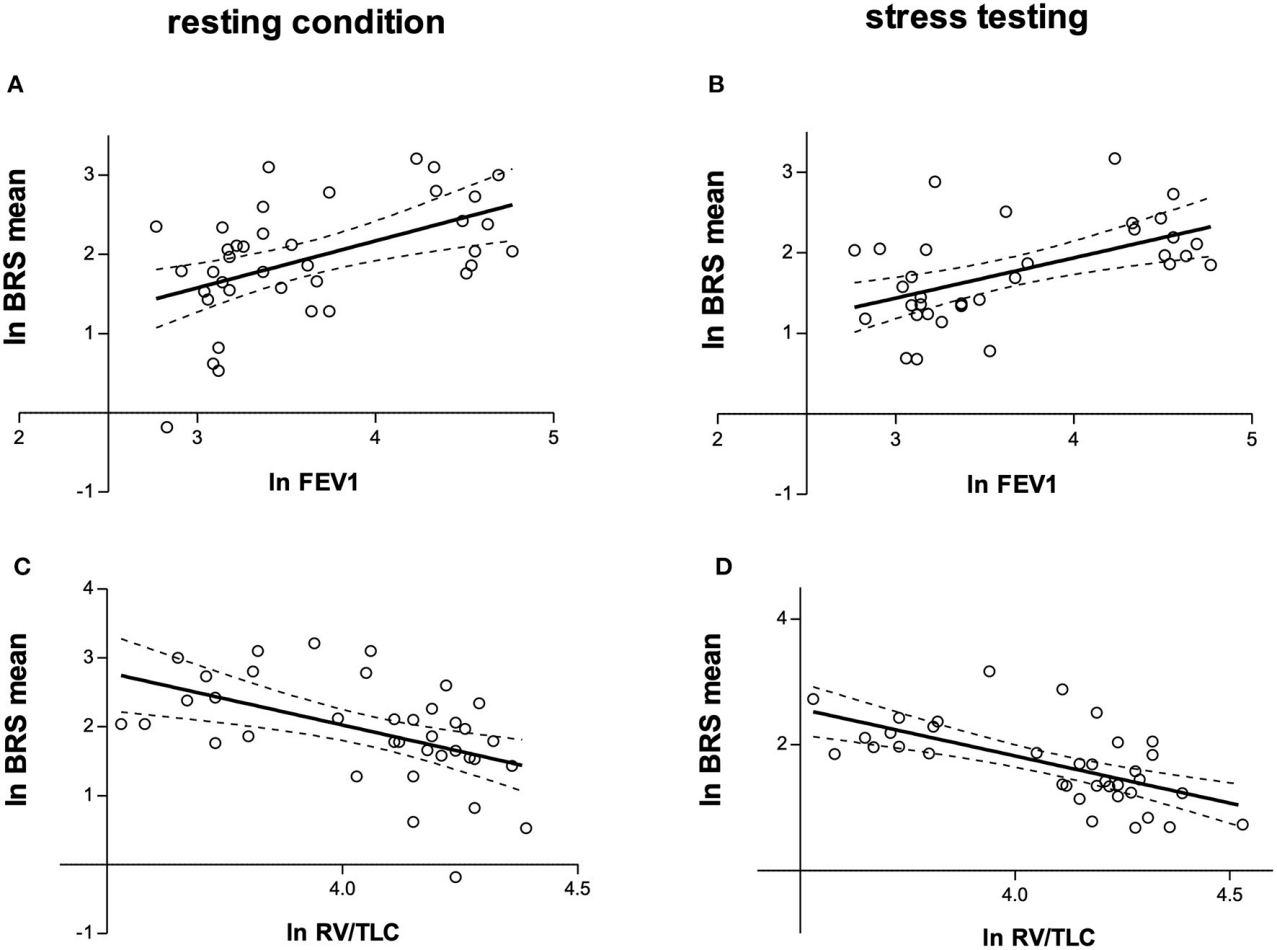
**(A)** Relationship between natural logarithms of FEV_1_% and BRSmean during resting condition (*r* = 0.496, *P* = 0.002). **(B)** Relationship between natural logarithms of FEV_1_% and BRSmean during mental arithmetic stress testing (*r* = 0.541, *P* = 0.001). **(C)** Relationship between natural logarithms of RV/TLC and BRSmean during resting condition (*r* = −0.496, *P* = 0.002). **(D)** Relationship between natural logarithms of RV/TLC and BRSmean during mental arithmetic stress testing (*r* = −0.624, *P* < 0.001). lnFEV1, natural logarithm of forced expiratory volume in one second predicted (FEV_1_%); lnRV/TLC, natural logarithm of residual volume/total lung capacity; BRS, baroreceptor reflex sensitivity; lnBRSmean, natural logarithm of the mean of BRSup and BRSdown.

Table 3 shows measurements of BRS in patients compared with controls, both during rest as well as stress testing. LnBRSup, lnBRSdown and lnBRSmean were significantly lower in patients with COPD than in controls, both during resting condition and mental arithmetic stress testing (Table 3, Figure 2). Mental arithmetic stress testing did not result in statistically significant changes in lnBRSmean, neither in patients nor in controls (*P* = 0.397; *P* = 0.142). By using parameters of airflow obstruction and hyperinflation as continuous variables, a significant correlation with lnBRSmean during rest (lnFEV_1_%: *r* = 0.496, *P* = 0.002; lnRV/TLC: *r* = −0.496, *P* = 0.002; Figures 3A,C) and stress testing (lnFEV_1_%: *r* = 0.541, *P* = 0.001; lnRV/TLC: *r* = −0.624*, P* < 0.001; Figures 3B,D) could be observed. An analysis of the patient group as a separate sample confirmed a significant correlation of lnBRSmean with lnRV/TLC (rest: *r* = −0.388, *P* = 0.050; stress: *r* = −0.428, *P* = 0.033), whereby the effect with lnFEV_1_% diminished (rest: *r* = 0.306, *P* = 0.121; stress: *r* = 0.090, *P* = 0.661) (Supplementary Figure 2). Similarly, there was a statistically significant relationship between SV during rest and lnBRSmean (*r* = 0.401, *P* = 0.013). In a multivariable regression analysis, using lnBRSmean as well as lnRV/TLC as independent variables, this relationship was not significant anymore (β = 0.161, *P* = 0.309). LnRV/TLC, however, was found to be a significant predictor of lnBRSmean, independent of heart rate during each condition (rest: β = −0.389, *P* = 0.037; stress: β = −0.443, *P* = 0.003). Furthermore, there was no relationship between lnBRSmean and arterial blood gases, neither with paO_2_ nor paCO_2_ (*P* > 0.1 for both).

**Table 3:** BRS slope values are indicated as geometric means with 95% CI.

	**COPD**	**Healthy subjects**	**Ratio of GM and 95% CI**	***P value***
**Resting conditions**				
BRSup, ms/mmHg	4.7 (3.4–5.9)	12.6 (8.9–18.6)	0.4 (0.2–0.6)	<0.001
BRSdown, ms/mmHg	6.2 (4.4–8.5)	10.9 (7.5–16.1)	0.6 (0.3–1.0)	0.034
BRSmean, ms/mmHg	5.6 (4.2–6.9)	12.0 (9.1–17.6)	0.5 (0.3–0.8)	0.003
**Stress testing**				
BRSup, ms/mmHg	4.1 (3.3–4.9)	9.0 (7.6–11.8)	0.5 (0.3–0.7)	<0.001
BRSdown, ms/mmHg	4.4 (3.4–5.5)	9.6 (6.9–15.5)	0.5 (0.3–0.7)	0.001
BRSmean, ms/mmHg	4.4 (3.7–5.3)	9.6 (7.7–12.2)	0.5 (0.3–0.7)	<0.001

*Differences are indicated as ratio of the geometric means with 95% CI*.

*COPD, chronic obstructive pulmonary disease; BRS, baroreceptor reflex sensitivity; BRSup, mean slope of sequences characterized by progressive increase in pulse interval and systolic blood pressure; BRSdown, mean slope of sequences characterized by progressive decrease in pulse interval and systolic blood pressure; BRSmean, mean of BRSup and BRSdown; GM, geometric mean; CI, confidence interval*.

## Discussion

We herewith provide evidence of impaired spontaneous BRS in patients with COPD. Furthermore, we were able to detect an independent relationship between hyperinflation and BRS in this report.

Although COPD is primarily defined as a chronic inflammatory disease of the lungs, it is accompanied by a variety of systemic features like cardiovascular comorbidities (1, 2). There is cumulating evidence of a direct effect of lung hyperinflation on cardiac function and diastolic filling (3–5). Consistent with these findings, we observed an inverse relationship between the severity of hyperinflation and stroke volume.

The autonomic nervous system with its baroreflexes ensures adaptation of blood pressure and heart rate and therefore stability of circulation during activities of daily living (22). BRS was shown to be an independent prognostic factor in patients after myocardial infarction, with 2-year mortality rates being significantly higher in those with a BRS below 3 ms/mmHg compared to those with higher values (9 vs. 2%) (10).

Few previous studies provided evidence of autonomic dysfunction in patients with COPD (6–9). Patakas et al. described an impairment of induced BRS after intravenous injection of phenylephrine in COPD patients compared to healthy controls. Patients had less severe airflow obstruction (mean FEV_1_% 38.5 ± 15.8) compared to those in the current report and a relationship of BRS with pulmonary function parameters was not tested (7). Bartels et al., similarly, observed lower BRS following Valsalva maneuver in COPD patients compared with healthy ex-smokers (9). While increases in intrathoracic pressure were speculated as a potential mechanism, the impact of lung hyperinflation was not tested in this report. Using a similar method of spontaneous BRS as in our report, Costes et al. studied the impact of an 8-week outpatient rehabilitation program on baroreflex activity in 21 patients with moderate COPD (23). BRS in patients was significantly lower than in controls, with modest improvements following the aforementioned exercise training program. The authors reported absence of a relationship between BRS and lung function tests, but unfortunately did not provide any data on this particular subject. Our findings extend the learnings from these earlier reports by applying a more rigorous patient selection to rule out concomitant cardiovascular disease, performing simultaneous and comprehensive assessment of beat-to-beat cardiovascular hemodynamic assessments, such as stroke volume, and performing lung function testing in both patients and controls and thus providing a wider range of parameters in order to test the initial hypothesis. Consequently, we were able to observe an independent relationship between parameters of hyperinflation and BRS. Multiple mechanisms may be responsible for these observations.

Valipour et al. previously demonstrated a reduction in spontaneous BRS following nasal CPAP induced increases in intrathoracic pressures in healthy volunteers (24). Pulmonary hyperinflation with distension of lung tissue and concomitant increases in intrathoracic pressures may possibly alter the sensitivity and activity of stretch-sensitive afferent vagal mechanosensors of the lungs (6, 25, 26). As their afferent inputs are known to modulate sympathetic responsiveness to arterial baroreceptor influences during normal respiration, chronic lung hyperinflation might result in an impairment of baroreflex pathways (6, 25, 26).

Furthermore, the presence of pulmonary hypertension may have a direct impact on baroreflex function as it has on cardiac filling (7, 27). Previous experimental studies provided evidence of an interaction between pulmonary arterial baroreceptors and systemic arterial baroreflexes (28). Patients in the current study, however, were free from overt pulmonary hypertension, thus above mechanism is rather unlikely to explain our observations.

Additionally, there is evidence of hypoxemia-driven alterations of baroreflex function (29, 30). In our study, we could not show any relationship between BRS and paO_2_. It needs to be acknowledged, however, that the overall level of hypoxemia in the present study was rather moderate and some of the patients received supplemental oxygen.

Our study has some limitations. First, the sample size is small. It was, both for patients and controls, not based on formal power calculation and, in the absence of appropriate data on hyperinflation and BRS from previous literature, chosen pragmatically to facilitate recruitment. Second, the study population is highly selected. We only studied emphysematous type of COPD patients without known relevant comorbidities, thus limiting the generalisability of our findings. We believe, however, that our homogenous patient selection contributes to ensure a more isolated observation of the interaction between pulmonary hyperinflation and BRS without other confounding factors. At the same time, we have to admit that both patients and controls may have had subclinical cardiovascular disease, which may have influenced our findings. Particularly, controls did not undergo in-depth diagnostic testing beyond lung function assessment, such as additional echocardiography or computed tomography, to rule out cardiopulmonary disease. However, in the absence of any medical history or symptoms of cardiopulmonary disease it is very unlikely that subclinical disease has significantly biased our findings. Furthermore, we did not observe any evidence of undiagnosed cardiovascular pathologies throughout the comprehensive cardiovascular testing performed during the study. Third, we have to acknowledge that the intended experimental stressor of mental arithmetic testing failed to result in substantial increases in heart rate. Thus, despite numerically lower values of BRS during stress testing, our findings cannot be automatically extrapolated to changing BRS during different external and internal stressors. Finally, we were not able to address the potential impact of inhaler therapy (e.g., bronchodilators) on BRS, as all cardiovascular assessments were done post-bronchodilator therapy. Nevertheless, since all COPD patients were at least on dual bronchodilator therapy, the relationship between hyperinflation and BRS appears to be independent of concomitant antimuscarinic or sympathomimetic influences. An important strength of this study, however, is the comprehensive, non-invasive hemodynamic measurement taken by validated testing methods.

Our results might have important therapeutic implications. Impairments in autonomic function may be—at least partially—reversible following pharmacological or non-pharmacological reductions in hyperinflation. In fact, improvements in cardiac function have previously been reported in response to volume reduction, by means of inhaled bronchodilators (31) as well as surgical (32) and bronchoscopic (33) lung volume reduction.

In conclusion, we were able to observe impaired BRS in patients with COPD and hyperinflation compared to healthy controls. Subsequent studies should examine whether lung volume reduction procedures are able to improve baroreceptor function and thus reduce cardiovascular risk in patients with COPD.

## Data Availability Statement

The original contributions presented in the study are included in the article/Supplementary Material, further inquiries can be directed to the corresponding author.

## Ethics Statement

Ethical approval was not provided for this study on human participants because according to regulations of the Ethics Committee of the City of Vienna, the Institutional Review Board approved the study. The patients/participants provided their written informed consent to participate in this study.

## Author Contributions

AM contributed to data analysis, statistical evaluations, interpretation and writing of the manuscript, and guarantees for its content. VW, SA, and IS contributed to subject recruitment, data collection, and investigation. G-CF contributed to data analysis and statistical evaluations. MU contributed to graphic design of figures. AV contributed to conceptualization, methodology, data analysis and interpretation, and writing of the manuscript. All authors contributed to manuscript revision, read, and approved the submitted version.

## Conflict of Interest

The authors declare that the research was conducted in the absence of any commercial or financial relationships that could be construed as a potential conflict of interest.

## Publisher's Note

All claims expressed in this article are solely those of the authors and do not necessarily represent those of their affiliated organizations, or those of the publisher, the editors and the reviewers. Any product that may be evaluated in this article, or claim that may be made by its manufacturer, is not guaranteed or endorsed by the publisher.

## Abbreviations

BMI: body-mass-index
BPdia: diastolic arterial blood pressure
bpm: beats per minute
BPmean: mean arterial blood pressure
BPsys: systolic arterial blood pressure
BRS: baroreceptor reflex sensitivity
BRSdown: mean slope of sequences characterized by progressive decrease in pulse interval and systolic blood pressure
BRSmean: mean of BRSup and BRSdown
BRSup: mean slope of sequences characterized by progressive increase in pulse interval and systolic blood pressure
CI: confidence interval
COPD: chronic obstructive pulmonary disease
DLCO: diffusion capacity of the lung for carbon monoxide
FEV_1_: forced expiratory volume in one second
FEV1%: forced expiratory volume in one second predicted
FRC: functional residual capacity
FVC: forced vital capacity
GM: geometric mean
ICS: inhaled corticosteroids
kg: kilogram
kPa: kilopascal
l: liter
LABA: long-acting beta agonists
LAMA: long-acting muscarinic antagonists
ln: natural logarithm
m_2_: square meter
min: minute
ml: milliliter
mmHg: millimeters mercury
mmol: millimole
ms: millisecond
paCO_2_: arterial partial pressure for carbon dioxide
paO_2_: arterial partial pressure for oxygen
PI: pulse interval
RV: residual volume
SI: stroke index
SV: stroke volume
TLC: total lung capacity.
