# Supplementary material for: Impaired Spontaneous Baroreceptor Reflex Sensitivity in Patients With COPD Compared to Healthy Controls: The Role of Lung Hyperinflation
Source: Front Med (Lausanne). 2022 Jan 3;8:791410. doi: 10.3389/fmed.2021.791410 (PMC8761648; doi:10.3389/fmed.2021.791410)
Supplement: Supplementary file 1 [file Table_1.pdf]

		lnFEV1%	RV	lnRV/TLC	TLC (l)	TLC (%)	lnBRSup (rest)	lnBRSdown (rest)	lnBRSmean (rest)
lnBRSup (rest)	Pearson's correlation coefficient	,551**	-,532**	-,587**	-,268	-,505**			
	P value	,000	,001	,000	,115	,002			
lnBRSdown (rest)	Pearson's correlation coefficient	,390*	-,304	-,347*	-,118	-,127			
	P value	,023	,085	,048	,514	,482			
lnBRSmean (rest)	Pearson's correlation coefficient	,496**	-,467**	-,496**	-,214	-,280			
	P value	,002	,004	,002	,204	,093			
lnBRSup (stress)	Pearson's correlation coefficient	,530**	-,441**	-,464**	-,224	-,405*			
	P value	,001	,007	,004	,190	,014			
lnBRSdown (stress)	Pearson's correlation coefficient	,493**	-,454**	-,605**	-,020	-,453**			
	P value	,002	,006	,000	,908	,006			
lnBRSmean (stress)	Pearson's correlation coefficient	,541**	-,520**	-,624**	-,122	-,520**			
	P value	,001	,001	,000	,477	,001			
SV	Pearson's correlation coefficient		-,435**	-,585**	-,030	-,573**	,502**	,346*	,401*
	P value		,004	,000	,849	,000	,002	,045	,013
SI	Pearson's correlation coefficient		-,440**	-,533**	-,118	-,403**			
	P value		,003	,000	,453	,007			

Table 1: Correlations between baroreceptor reflex sensitivity, SV, SI and parameters of lung function. Legend: ln: natural logarithm; BRSup: mean slope of sequences characterized by progressive increase in pulse interval and systolic blood pressure; BRSdown: mean slope of sequences characterized by progressive decrease in pulse interval and systolic blood pressure; BRSmean: mean of BRSup and BRSdown; SV: stroke volume; SI: stroke index; FEV_1%: Forced expiratory volume in one second predicted; RV: Residual volume; TLC: Total lung capacity.
