# Supplementary material for: Impaired Spontaneous Baroreceptor Reflex Sensitivity in Patients With COPD Compared to Healthy Controls: The Role of Lung Hyperinflation
Source: Front Med (Lausanne). 2022 Jan 3;8:791410. doi: 10.3389/fmed.2021.791410 (PMC8761648; doi:10.3389/fmed.2021.791410)
Supplement: Supplementary file 2 [file Image_1.pdf]

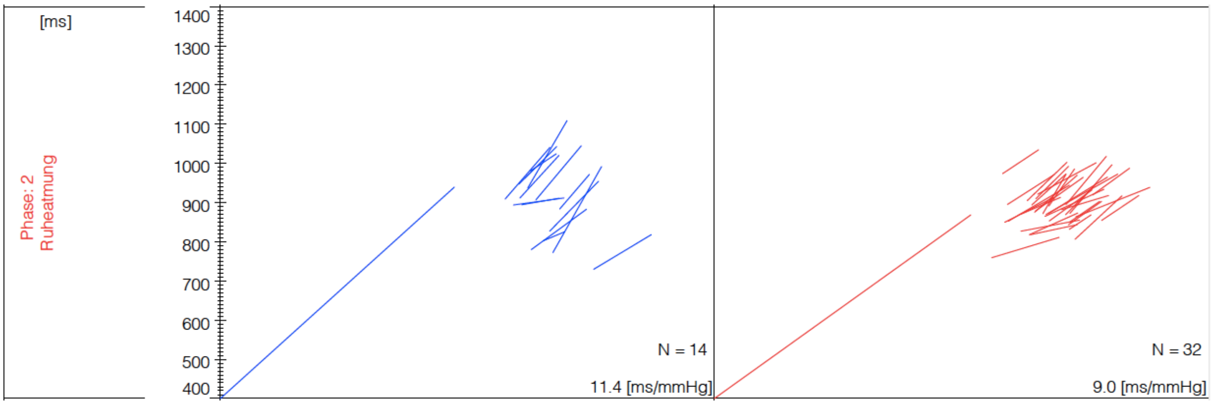

Figure 1: Measurement of BRS during rest. Blue regression lines indicating up-sequences, red regression lines down-sequences. The mean slopes from all regression lines are indicated at the lower left corner and represent BRSup or BRSdown, respectively. Legend: BRS: Baroreceptor reflex sensitivity
