# Supplementary material for: Impaired Spontaneous Baroreceptor Reflex Sensitivity in Patients With COPD Compared to Healthy Controls: The Role of Lung Hyperinflation
Source: Front Med (Lausanne). 2022 Jan 3;8:791410. doi: 10.3389/fmed.2021.791410 (PMC8761648; doi:10.3389/fmed.2021.791410)
Supplement: Supplementary file 3 [file Image_2.pdf]

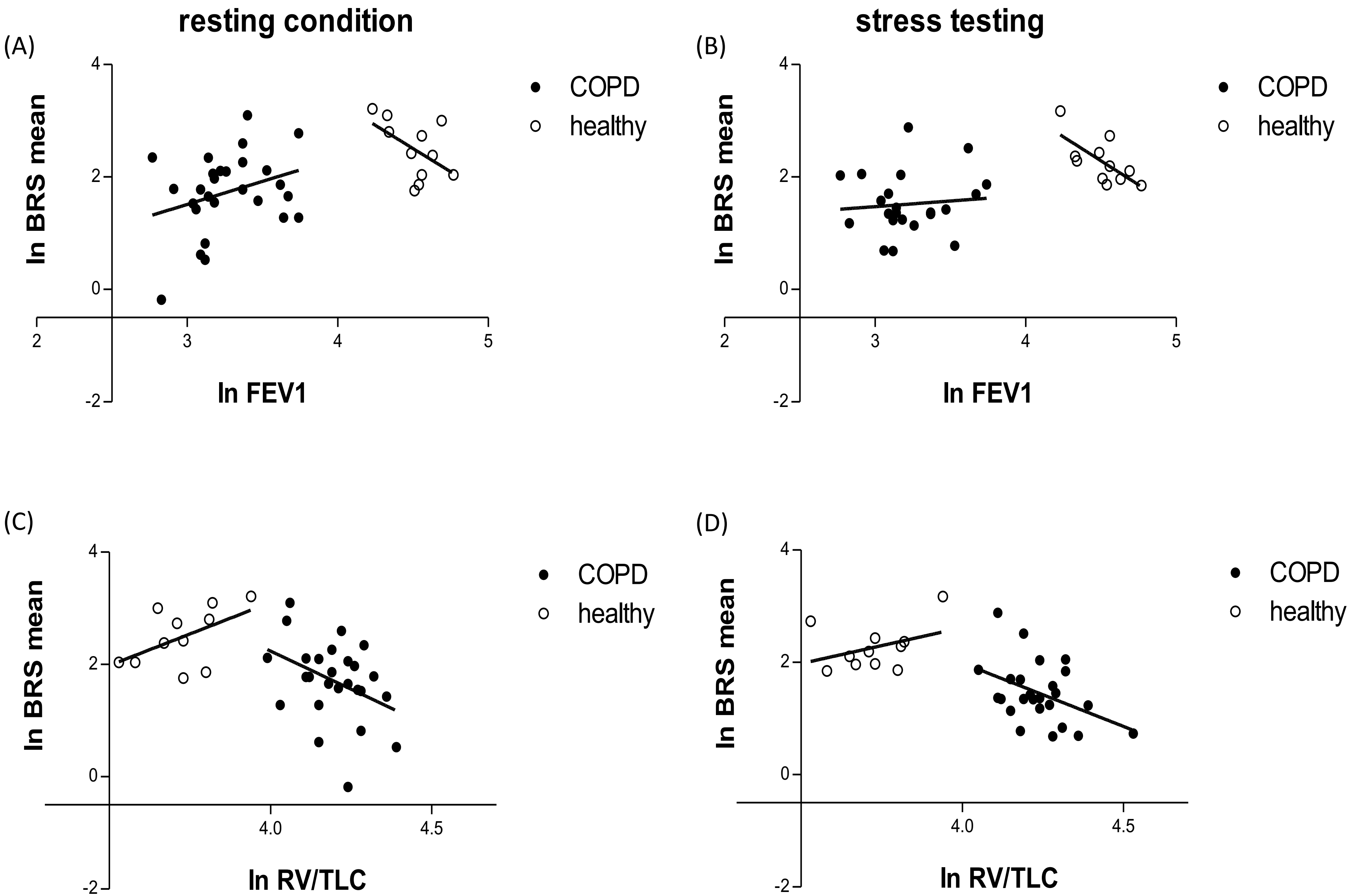

Figure 2: **(A)** Relationship between natural logarithms of FEV_1% and BRSmean during rest, COPD (r=0.306, *P*=0.121), healthy (r=-0.518, *P*=0.103). **(B)** Relationship between natural logarithms of FEV_1% and BRSmean during stress, COPD (r=0.090, *P*=0.661), healthy (r=-0.682, *P*=0.021). **(C)** Relationship between natural logarithms of RV/TLC and BRSmean during rest, COPD (r=-0.388, *P*=0.050), healthy (r=0.506, *P*=0.112). **(D)** Relationship between natural logarithms of RV/TLC and BRSmean during stress, COPD (r=-0.428, *P*=0.033), healthy (r=0.361, *P*=0.276). Legend: ln FEV1: natural logarithm of forced expiratory volume in one second predicted; ln RV/TLC: natural logarithm of residual volume/total lung capacity; BRS: baroreceptor reflex sensitivity; lnBRSmean: natural logarithm of the mean of BRSup and BRSdown
